# An Integrative Behavioral Model of Information Security Policy Compliance

**DOI:** 10.1155/2014/463870

**Published:** 2014-05-28

**Authors:** Sang Hoon Kim, Kyung Hoon Yang, Sunyoung Park

**Affiliations:** ^1^Department of Business Administration, Kwangwoon University, 26 Kwangwoon-gil, Nowon-gu, Seoul 139-701, Republic of Korea; ^2^College of Business School, University of Wisconsin-La Crosse, 1725 State Street, La Crosse, WI 54601, USA; ^3^Namyang R&D Center, Hyundai Motor Company, 772-1 Jangdeok-dong, Hwaseong-Si, Gyeonggi-do 445-706, Republic of Korea

## Abstract

The authors found the behavioral factors that influence the organization members' compliance with the information security policy in organizations on the basis of neutralization theory, Theory of planned behavior, and protection motivation theory. Depending on the theory of planned behavior, members' attitudes towards compliance, as well as normative belief and self-efficacy, were believed to determine the intention to comply with the information security policy. Neutralization theory, a prominent theory in criminology, could be expected to provide the explanation for information system security policy violations. Based on the protection motivation theory, it was inferred that the expected efficacy could have an impact on intentions of compliance. By the above logical reasoning, the integrative behavioral model and eight hypotheses could be derived. Data were collected by conducting a survey; 194 out of 207 questionnaires were available. The test of the causal model was conducted by PLS. The reliability, validity, and model fit were found to be statistically significant. The results of the hypotheses tests showed that seven of the eight hypotheses were acceptable. The theoretical implications of this study are as follows: (1) the study is expected to play a role of the baseline for future research about organization members' compliance with the information security policy, (2) the study attempted an interdisciplinary approach by combining psychology and information system security research, and (3) the study suggested concrete operational definitions of influencing factors for information security policy compliance through a comprehensive theoretical review. Also, the study has some practical implications. First, it can provide the guideline to support the successful execution of the strategic establishment for the implement of information system security policies in organizations. Second, it proves that the need of education and training programs suppressing members' neutralization intention to violate information security policy should be emphasized.

## 1. Introduction

These days, many corporations are beginning to recognize that technology-based solutions alone cannot reduce security risks; therefore, they are beginning to emphasize the managerial factors of security as well as technological and physical ones. As managerial issues have become important, the members' compliance with the information security policy in organizations emerges as a core issue of the managerial issues [1, 2]. Even though organizations provide and support the policy and education, in order for the policies and education to be effective, each member should comply with the actual security guidance and observance of the policy.

In the paper, the authors researched to find the factors for organization members to comply with the information security policy. For that purpose, a model which was based upon the related theories was suggested and validated. The theoretical implications of this study are as follows: the study is expected to play a role of the baseline for future research about organization members' compliance with the information security policy, the study attempted an interdisciplinary approach by combining psychology and information system security research, and the study suggested concrete operational definitions of influencing factors for information security policy compliance through a comprehensive theoretical review. The derived results could be applied to build the strategy and the future research issues will be discussed as well.

## 2. Literature Review

### 2.1. Compliance with the Information Security Policy

Many security experts use technical terminologies such as error, malfunction, breach, or failure when they explain security problems. That means that security problems are traditionally considered to be technology problems and more advanced algorithms or technologies are suggested to solve security problems. As a consequence, the more advanced security-threating technologies emerge, the more experts try to develop further advanced security technologies to nullify the new threating technologies. It is a cycle where continually arising security threats create the necessity for more advanced security techniques. This can be a waste of time and effort and, furthermore, it cannot be an ultimate solution to security. Therefore, to disconnect this endless circulation, a different approach, one that is managerially as well as technically efficient, should be considered. As managerial aspects are emphasized, members' compliance with the information security policy becomes a hot issue. Bulgurcu et al. [3] mentioned that the main stream of research of human perspective of information security is to find the factors that connect the end users' behaviors and members' compliance with the information security policy in organizations. Mistakes, errors, inappropriate usage, and ignorance of the members make the information security system of the organization dangerous [4]. Therefore, it has been recognized that appropriate knowledge and activities are the most important factors for the information systems security in the organizations. For that purpose, the most important factor is the members' compliance with the information security policy in organizations.

### 2.2. Theory

To build the model, instead of one main theory, four theories are used: planned action theory, rational choice theory, neutralization theory, and protection motivation theory. Even though the four theories were developed independently, all four explain the behavior of the users and all of them are harmonious in explaining security policy compliance. The research model was built based upon the four theories mentioned above.

#### 2.2.1. Planned Action Theory and Theory of Planned Behavior

Traditionally, attitude has been considered a major variable in explaining the actions of human in social psychology. However, many scholars have insisted that other factors besides attitude should be also considered to explain the relationship among attitude, intentions of action, and action. One of the theories is the theory of reasoned action (TRA) which was suggested by Fishbein and Ajzen [5]. However, this theory's validity and the limits of the applications have been criticized. This theory assumes that each individual can control one's actions, but in the real world, there are many cases where an individual cannot control his or her actions [6]. Even though they may have a positive attitude and subjective norms, an individual cannot carry the action when they do not have opportunities and resources. Therefore, action control factors should be added to TRA to cover the limits. The extended theory is the theory of planned behavior (TPB) which was suggested by Azjen [7]. According to the theory of planned behavior, an individual will perceive the fact that they can control the actions only when they have a positive attitude and subjective norms towards the actions as well as opportunities and resources of the actions, and they will begin to have an intention of action only when they perceive the fact that they can control it.

#### 2.2.2. Rational Choice Theory

Bulgurcu et al. [3] had researched the topic of members' compliance with the information security policy in organizations on the basis of the rational choice theory and perception of information security. The rational choice theory insists that an individual makes a decision by comparing the costs and benefits of one's decision making. The rational choice depends on the perceptions of the individual.

#### 2.2.3. Neutralization Theory

Many researchers have suggested sanctions to security policy violators such as penalties and punishments based on the control theory [8–11]. Although control theory could explain the reason for security policy violations, the explanation of these theories is limited, because the potential factors of security policy violence are not clearly identified and these theories cannot be used to prevent violence.

Siponen and Vance [12] insisted that the fear of punishment cannot explain the policy violence actions because members also know and apply the theory to their actions. The Neutralization theory was first suggested by Piquero et al.  [13] to explain crimes in criminology. The theory insists that both those that obey the rules and those that violate them respect the norms and values of the community. In spite of this, the actual reason that some people violate the rules, according to the neutralization theory, is that they somehow justify themselves [14]. Rogers and Buffalo [15] insisted that the neutralization theory is the theory that explains how people nullify the existing norms of society by justifying the violation of the norm. Sykes and Matza [14] suggested the five types of the neutralization techniques that justify the offenders. The first is the denial of responsibility with which the violator denies responsibility [14, 15]. The second is the denial of injury. Here, they insist that what they did was the best way to minimize injury to the organization. The third is the way in which it appeals to the higher loyalties of the organization. It means that the offenders admitted that they were wrong; however, they insist that their actions were performed in order to protect or support organizations such as their family, friends, or company. The fourth is the condemnation of the condemners. This means that the violators countercondemn the people who condemn the violators and neutralize their activities. The fifth is the denial of wrongdoing to the victim. They believe the victims deserved to be punished. This mentality is usually used to justify attacks on members of minority groups, such as homosexuals [16].

Siponen and Vance [12] studied the relationship between the neutralization theory and the violation of information security policy in organizations. In their research, they deleted “the denial of injury” which was suggested by Sykes and Matza [14] and added “the metaphor of the ledger” which was suggested by Klockars [17] and “the defense of necessity” which was suggested by Minor [18]. The basic idea of the metaphor of the ledger is that individuals think that any good deeds they have performed should outweigh a few harmful actions [13, 17]. In the defense of necessity point of view, violators believe that it is unnecessary to feel guilty as rule violations are sometimes unavoidable in life [18].

Cressey [19] introduced “the defense of ubiquity” and it was further developed by Coleman [20]. The main idea of this concept is that violators justify their activities by insisting that almost everybody commits those kinds of violations of norms. Therefore, there is no need to feel guilty.

#### 2.2.4. Protection Motivation Theory

The protection motivation theory explains how individuals change their attitudes and actions when facing danger. This theory was developed by Rogers [21]. The theory, mainly developed in the field of psychology, tries to find the factors that affect the intentions of activity based upon “fear appeal.” According to the theory, when an individual is exposed to a message of danger, protection motivations that stimulate the actions are made. This theory assumes that there are three factors in fear appeal: the severity which measures the extent of the threat, the exposure which measures the possibility of being exposed to the threats, and the response efficacy which measures how to treat the threats efficiently. Later, Rogers [22] added self-efficacy to the list.

Johnston and Warkentin [23] studied the relationship between security activity and the fear appeal and derived the research model based upon the protection motivation theory. They also added “the social effects” and “the intentions of action” which were used in technology adoption. Furthermore, they assumed that severity and danger sensitivity affected efficacy and efficacy directly affected intentions of behavior.

## 3. Research Model and Hypotheses

**Figure 1:**
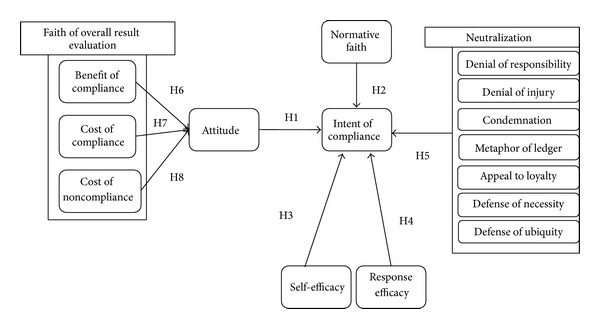
Research Model.

The research assumed that the attitudes, the norms, and the self-efficacy of members affected the intentions of information security policy compliance based upon the theory of reasoned action and the neutralization theory. Also based upon the research of Ajzen [7] and Bulgurcu et al. [3], the authors assumed that the belief towards information security policy compliance affected the attitude of information security policy compliance. Also, the authors assumed that the response efficacy affected the intentions of information security policy compliance based upon the protection motivation theory. Based upon the previous research and the above assumptions, the integrated behavioral research model, as described in Figure 1, was suggested.

Three independent variables, the attitude, the subjective norm, and the perceived control of actions, have been derived upon the theory of reasoned action to measure the intentions of action. In the research, the meanings of the variables are modified without loss of generality. A variable “attitude” means the attitude towards the security policy compliance, and “normative belief” means the normative belief of security policy compliance. Also, Ajzen [7] found that the “perceived action control” of the theory of reasoned action derived from the concept “self-efficacy,” with the meaning of these two concepts (perceived action control and self-efficacy) being similar. In the research, based upon the research of Ajzen [7], the concept of self-efficacy towards security policy compliance implies the concept of perceived action control.

Based upon the theory of reasoned action, the following three hypotheses are set up.

*Hypothesis  1.* The more positive the attitude of the members of the organization towards information security policy compliance, the higher the intention of information security policy compliance.

*Hypothesis  2.* The stronger the normative belief of the members of the organization towards information security policy compliance, the higher the intention of information security policy compliance.

*Hypothesis  3.* The stronger the self-efficacy of the members of the organization towards information security policy compliance, the higher the intention of information security policy compliance.

“Response efficacy” means the degree of individual belief that the recommended plans of action to the threats are effective [21, 24]. According to the protection motivation theory, response efficacy has positive effects in the decrease of threats by adopting the recommended plans of action. By applying the above theory to the research, the information security policy can be considered as the recommended plan of action to the threats. Therefore the degree of belief that the information security policy will be effective towards information security can be considered as a response efficacy. The higher the degree of which the members of the organization believe that the information security policy to information security is effective, the higher the intentions of information security policy compliance. Based upon the above inference, Hypothesis*  *4 is set up.

*Hypothesis  4.* The stronger the response efficacy of the members of the organization towards information security policy compliance, the higher the intention of information security policy compliance.

According to previous research, people have the intention to present themselves in an amicable image [25, 26]. Therefore, when a member of the organization commits a wrongdoing, he or she tries to justify their action and uphold their image. The neutralization theory explains how the members of an organization excuse and justify their unjustified behaviors. The neutralization theory was used in the research to explain how the members of an organization justify the violation of information security policy.

There are critics that say that the neutralization theory was developed to explain crimes such as felonies or misdemeanors and that applying this theory to the violation of information security policy is not appropriate. However, even though the violation of the information security policy is not a crime, both are violations of the social norms. Akers and Sellers [27] insisted that a violation can be applied to social norms as well as crimes. Based upon previous research [27], Siponen and Vance [12] applied the neutralization theory to the information security policy.

In the research, the neutralization theory is analyzed by the second-order construct, which consists of several subfactors. The reason of the second-order construct is that the neutralization theory consists of several dimensions, and that should be represented in the modeling [28]. Furthermore, the basic factors are already found in the previous research [12]. Hypothesis*  *5 is derived based upon the above reasoning.

*Hypothesis  5.* The higher the neutralization levels of the members of the organization, the lower the degree of information security policy compliance of the members of the organization.

According to previous research, the individual attitude towards the action is related to the individual belief towards the results of the action [5, 7]. Also, according to the rational choice theory, the individual considers the cost and benefits of the action and decides which has a larger net benefit. In the research, variables “attitude” and “belief” are brought from the rational choice theory. It means that the member will consider the cost and benefit and will decide whether he will violate the norm. For the belief of the overall evaluations, three variables are considered: benefit of compliance, cost of compliance, and cost of noncompliance. The attitude of the members would be more favorable towards the information security policy compliance when the benefit of compliance is bigger than the cost of compliance or the net benefit of the noncompliance. This assumption is in accord with previous research that insists there is a positive relationship between policy compliance and the judgment made by the cost benefit analysis of policy compliance [29]. Furthermore, Price Waterhouse Coopers [30] had similar results where there was a negative relationship between the intentions of the policy compliance and the cost of the policy compliance. Based upon previous research, the following hypotheses are derived.

*Hypothesis  6.* The higher the degree of the perception of benefit by the organization members towards information security policy compliance, the higher the intention of information security policy compliance.

*Hypothesis  7.* The higher the degree of the perception of cost by the organization members towards information security policy compliance, the lower the intention of information security policy compliance.

*Hypothesis  8.* The higher the degree of the perception of cost by the organization members towards information security policy noncompliance, the higher the intention of information security policy compliance.

## 4. Construction of Variables and Measurement

### 4.1. Construction of Variables

The majority of the variables used in the research came from previous research and some of them have been modified for the purpose of this research. An advantage of using variables from previous research is that the variables have already been verified. The variables are summarized in Table 1. Based upon these definitions, Likert scale-based measure indices were made.

### 4.2. Data Collection and Analysis

The authors surveyed the information systems users in the organizations that have information security policies. They reviewed a long list of companies that were then randomly selected. The surveyors visited the companies and explained the purposes of the research. Eventually 32 companies from 10 industries were randomly selected. Two or three people from each rank, as well as the line worker, middle manager, and top manager in each company, answered the questionnaires. The distribution of the response of rank is 26 top managers (13.4%), 85 middle managers (43.8%), 68 line workers (35.1%), and 15 no responses (7.7%). The distribution of age is 75 in their 20's (38.7%), 61 in their 30's (31.4%), 39 in their 40's (20.1%), 14 in their 50's (7.2%), and 5 no responses (2.6%).

To measure the “neutralization,” the scenario method was used. Based upon the Siponen and Vance [12] research, the scenario cases were prepared. Three scenarios were prepared and a scenario was randomly selected. The selected scenario was included in the questionnaires. After reading the scenario on the violation of the information security compliance that had happened in other companies or had a possibility of happening, the respondents answered the questionnaires. The reason that a scenario method was used is because if the questions were asked directly, then the respondents might not be willing to answer the questionnaires frankly or might answer according to perceived social norms when asked about ethical issues such as violations against the information security compliance. The advantage of the scenario method is that the respondents can answer without any guilt or fear of exposing oneself because the scenario method assumes that the case is not real or has happened to someone else [31].

A total of 207 questionnaires were collected and 13 were excluded due to insincere or incomplete answers and 194 questionnaires were used. The survey took approximately three months. The descriptive explanation is summarized in Table 2.

## 5. Hypotheses Test and Analysis of Results

For the test of reliability and validity of variables, the structural equation modeling (SEM) was used. The software SPSS 18.0 was used for the data analysis. The PLS method was used for the reliability, validity, and hypotheses tests. PLS is known to be suitable in analyzing relatively small size data. Furthermore, LISEL is not as accurate when two level analyses are used, such as the neutralization of this research [32]. By considering the above factors, “SmartPLSver. 2.0. M3” was adopted.

### 5.1. Reliability and Validity of Variables

#### 5.1.1. Reliability Test

According to previous research [32], when the composite reliability (CSRI) is above 0.7 and the average variance extracted (AVE) is above 0.5, the variables are considered to be internally consistent. In the research, the AVE of every variable is above 0.73, the CSRI is above 0.95, and Cronbach's alpha is also above 0.93. Therefore, the data are considered to be reliable.

#### 5.1.2. Validity Test

It is recommended that the value of factor loading should be above 0.7 and the value of factor loading should be greater than that of the cross loading in order to have a convergent validity [33, 34]. In this research, every condition is satisfied and the result is summarized in Tables 3 and 4. To measure the discriminant validity, the values of factor loading are compared to the values of cross loading and it was found that the former is greater than the latter.

The second condition for the discriminant validity is that the square root of the values of the average variance extracted (AVE) should be bigger than the correlation coefficient. In this research, this condition is also satisfied. Therefore, the discriminant validity is satisfied.

### 5.2. The Fitness Test of the Model

Since the objective of PLS analysis is to maximize the variance explained, and assumptions regarding the distribution are not set up in PLS analysis, the fitness test among the explained variance of endogenous variables is preferred rather than the goodness-of-fit measures in covariance structure analysis. Therefore the forecasting fitness and the goodness-of-fit should be considered. For the forecasting fitness, *R* ^2^ is used. The range of high (above 0.26), middle (between 0.13 and 0.26), and low (above 0.02 and below 0.13) is used for classification [32]. However, *R* ^2^ is not good for a convenient measure. Therefore the value of the redundancy value of the Stone-Geisser Q2 test is more popularly used [35]. If the value of redundancy is greater than 0, it is interpreted to be forecasting fitted. Goodness-of-fit is calculated by the square root of value which is obtained by multiplying the average of *R* ^2^ and the average of communality [36]. The value of the degree of fitness should be greater than 0.1 and classified as higher (above 0.36), middle (0.25~0.36), and lower (0.1~0.25). The result is summarized in Table 5. In Table 5, *R* ^2^ of compliance intentions is 81.6% and that of attitude is 61.1%; the values of redundancy are positive numbers. The goodness-of-fit measure of the model is 0.857, which is significant. Overall, the model passes the goodness-of-fit measures test.

### 5.3. Results of the Hypotheses Test and Discussion

**Figure 2:**
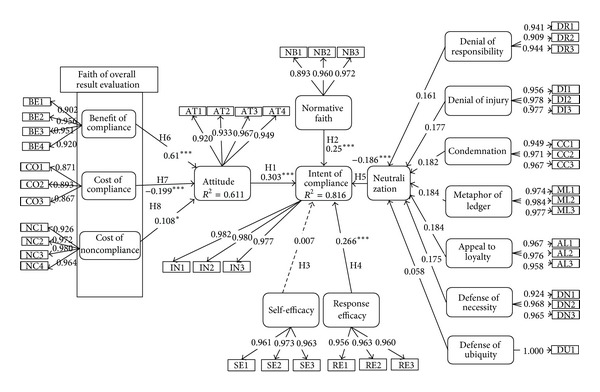


The bootstrap method was used to evaluate the path coefficient because PLS cannot show the significance of the path coefficient and the confidence level. The results are summarized in Figure 2.

*Hypothesis  1.* The path coefficient of Hypothesis*  *1 is 0.303 and the *t* value is 3.895; therefore, this hypothesis is supported. This means that the theory of reasoned action is confirmed to be appropriate in explaining the attitude towards the information security as previous research insisted. Therefore, we can say that the more positive the attitude of the members of the organization towards information security policy compliance, the higher the intention of information security policy compliance. Based upon this research result, policy makers should make a policy which can more positively develop the attitude of the members of the organization towards information security policy compliance.

*Hypothesis  2.* Hypothesis*  *2 is supported. The path coefficient of Hypothesis*  *2 is 0.25 and the *t* value is 3.136. The result says that when the members of the organization comply with the information security policy, they consider not only their internal factors but also their external environment as well as social factors. The environmental and social factors in the organization include the supervisors, colleagues, and top managers. Therefore, the relationship with them will affect the intentions of the information security police compliance. 

*Hypothesis  3.* The path coefficient of Hypothesis*  *3 is 0.007 and the *t* value is 0.128 which means that there is no statistical significance between the two variables. Hence this hypothesis is rejected. Based on the research, there is no relationship between self-efficacy of the members of the organization towards information security policy compliance and the intention of information security policy compliance. This can be interpreted as the higher self-efficacy of the members of the organization towards information security policy compliance not affecting the intentions of information security policy compliance of the members of the organization. This result is different from previous results [7] and further research is required. 

*Hypothesis  4.* The path coefficient of Hypothesis*  *4 is 0.266 and the *t* value is 4.385, so this hypothesis is supported. This result means that the more the members of an organization consider the information security policy to be effective, the more their intentions of compliance will increase. Therefore, it is important to make the members of an organization believe that the information security policy is effective. 

*Hypothesis  5.* Hypothesis*  *5 is supported because the path coefficient is −0.186 and the *t* value is 4.552. The results can be interpreted as the members of the organization try to justify the violation of information security policy compliance through the seven types of neutralization techniques mentioned in the research. The one thing to emphasize is that all of seven types of neutralization techniques are found to be significant and all of them should be considered. 

*Hypothesis  6.* The path coefficient and *t* value are 9.181 and 0.61, respectively, and this hypothesis is supported. The result says that the benefit of compliance is higher than the cost of benefit or cost of noncompliance. It means that the benefit of compliance has a higher influence than the cost of benefit or cost of noncompliance to the information security policy compliance. The benefit of compliance can include financial benefits, reputations, and positive factors for promotions, satisfaction, and pride. Therefore, policy makers should consider these factors to increase the benefit of compliance to make the members comply with the information security policy. 

*Hypothesis  7.* Hypothesis*  *7 is supported because the path coefficient and the *t* value are 3.699 and −0.199, respectively. The results say that the more the members of the organization recognize the cost of compliance, the more the members of the organization do not comply with the information security policy. The cost of compliance can include annoyances, time effort and so on. Therefore, policy maker should consider these factors in decreasing the cost of compliance to make the members comply with the information security policy. 

*Hypothesis  8.* The path coefficient and *t* value are 0.108 and 1.716, respectively, and the hypothesis is supported. The results say that the more the members of the organization recognize the cost of noncompliance, the more the members of the organization comply with the information security policy. The cost of noncompliance can include guilt, bad reputations, and disadvantages of promotion. Therefore, policy makers should consider these factors in increasing the cost of noncompliance to make the members comply with the information security policy.

## 6. Conclusion and Limits

### 6.1. Contribution

In the research, the authors try to find the factors of information security policy compliance and suggest the information security policy based upon the founded factors. For those purposes, the authors reviewed the previous research and the related literature. They reviewed the concept of information security and security policy. After that, based upon the literature review, they derived the factors which affect the intentions of policy compliance. In detail, they derived attitude, normal belief, and self-efficacy based upon the theory of reasoned action, seven factors from the neutralization theory, and response efficacy from the protection motivation theory. Based upon the mentioned theory, they set up the model and hypotheses, analyze, and found the seven out of eight hypotheses to be supported.

### 6.2. Limitations

This paper also has the following limitations. The first is the application of multiple theories. By using multiple theories, coordination can be an issue. However, the authors tried to find the common factors of the theory for the one subject. The second is the collection of data. Because the authors collected the companies that have security, it is possible that the data collection is biased. Lastly, there are factors other than the behavioral effects affecting the security. Those factors should also be considered in future research.

In spite of the above limits, the research has the following theoretical implications. First, the authors found the factors based upon the several previous theories, and they expect that these founded factors could be used as the factors towards the intentions of information security policy compliance for future research. Second, they adopted several theories from several arenas such as neutralization theory in criminology and combined them with the theories in the information systems field. Third, they defined the construction of the factors which were obtained through literature. These variable constructions would be used for future research in the area of information security policy.

### 6.3. Implications

The practical implications of this research are as follows. First, the result of the research can be used as the guideline for the practitioners. Second, the research shows that there is a positive relationship between the members' belief of security policy effectiveness and policy compliance. Therefore this research would provide the theoretical foundations for the cost and benefit of the policy compliance. Therefore, the more the members trust security policy, the more the members comply with the policy. As such, this finding can be used as the foundation of security policy education. Third, the research found that neutralization weakens the intentions of the policy compliance, and, therefore, the organization needs some training programs or education which oppresses neutralization. Fourth, the research found that response efficacy affects the intentions of policy compliance but self-efficacy does not. It implies that the education program which enlightens members that the security policy is actually effective to the organization security is better than the reinforcement of techniques of the members to security.

## Figures and Tables

**Table 1:** Definition of variables.

Variables	Definition	Items	Related research
Intentions of compliance	The degree of intentions which protects the information and resources of the organization from potential threats by the compliance of information security policy	IN1~3	Bulgurcu et al. [3]

Normative belief	The degree of perceptive social pressure of neighbors such as the supervisor, colleague, and manager when they comply with the policy	NB1~3	Bulgurcu et al. [3]

Neutralization	The degree of logic which nullifies the existing norm of society that is related to the compliance of information security policy by justifying the violation of the norm.		Sykes and Matza [14] Siponen and Vance [12] S. J. Lee and M. J. Lee [16]
Neutralization theory			
Denial of responsibility	The degree that the violator denies responsibility of the compliance violation of the information security policy	DR1~3	
Denial of injury	The degree that what they did was the best way to minimize the injury of the compliance violation of the information security policy.	DI1~3	
Appeal to higher loyalties	The degree that they believe there was no other way to protect their groups except through the compliance violation of the information security policy.	AL1~3	
Condemnation of condemners	The degree that the violators condemn the condemners to neutralize the compliance violation of the information security policy.	CC1~3	
Metaphor of the ledger	The degree of belief that the compliance violation of information security policy would be accepted because of the many good deeds that they have done in the past.	ML1~3	
Defense of necessity	The degree that there is no need to feel guilty for the compliance violation of the information security policy because the violation was unavoidable.	DN1~3	
Defense of ubiquity	The degree that the violators justify the compliance violation of the information security policy by insisting that almost everybody violates policies.	DU1	

Attitude	The degree to which compliance of information security policy affects the evaluation positively	AT1~4	Bulgurcu et al. [3]

Benefit of compliance	The degree of the perception of benefit by the members of the organization towards information security policy compliance	BE1~4	Bulgurcu et al. [3]

Cost of compliance	The degree of the perception of cost by the members of the organization towards information security policy compliance	CO1~3	Bulgurcu et al. [3]

Cost of noncompliance	The degree of the perception of cost by the members of the organization towards information security policy noncompliance	NC1~4	Bulgurcu et al. [3]

Self-efficacy	The degree of the individual's confidence that they have enough techniques, knowledge, and ability on the information security policy	SE1~3	Bulgurcu et al. [3]

Response efficacy	The degree of belief that the information security policy can handle the threats efficiently	RE1~3	Johnston and Warkentin [23]

**Table 2:** Demographic characteristics of the respondents.

Classification		Frequency	Percentage (%)
Gender	Male	127	65.5
	Female	67	34.5

Age	20~29	75	38.7
	30~39	61	31.4
	40~49	39	20.1
	50~59	14	7.2
	No response	5	2.6

Education	High school graduate	23	11.9
	Undergraduate	144	74.2
	Graduate school	27	13.9

Number of employees	<100	73	37.6
	100~1,000	61	31.4
	1,000~10,000	32	16.5
	>10,000	8	4.1
	No response	20	10.3

Annual sales volume	<$10 M	49	25.3
	$10 M~$100 M	33	17.0
	$100 M~$1000 M	28	14.4
	$1000 M~$1 B	20	10.3
	>1 B	2	1.0
	No response	62	32

Category of business	Manufacturing	33	17
	Construction	12	6.2
	Communication	30	15.5
	Transportation	1	0.5
	Distribution	12	6.2
	Service	55	28.4
	Finance	30	15.5
	And so forth	21	10.8

Rank	Line worker	68	36.4
	Middle manager	57	30.5
	General Manager	28	15.0
	CEO	26	13.9
	And so forth	8	4.3
	No response	7	3.6

Department	Planning/Administration	39	20.1
	Personnel/Education	14	7.2
	R&D	22	11.3
	Marketing	37	19.1
	Computer/Information	55	28.4
	And so forth	23	11.9
	No response	4	2.1

**Table 3:** Factor loading and cross loading.

Variables	Appeal to higher loyalties (AL)	Attitude (AT)	Benefit of compliance (BE)	Condemnation of condemners (CC)	Cost of compliance (CO)	Denial of injury (DI)	Defense of necessity (DN)	Denial of responsibility (DR)
AL1	**0.967**	−0.591	−0.431	0.823	0.528	0.756	0.818	0.725
AL2	**0.976**	−0.596	−0.429	0.812	0.508	0.747	0.826	0.729
AL3	**0.958**	−0.571	−0.394	0.804	0.547	0.724	0.842	0.712
AT1	−0.641	**0.920**	0.640	−0.593	−0.493	−0.645	−0.604	−0.656
AT2	−0.499	**0.933**	0.737	−0.467	−0.415	−0.523	−0.457	−0.576
AT3	−0.574	**0.957**	0.714	−0.491	−0.499	−0.580	−0.540	−0.620
AT4	−0.560	**0.949**	0.747	−0.527	−0.456	−0.553	−0.523	−0.614
BE1	−0.484	0.734	**0.902**	−0.457	−0.422	−0.581	−0.450	−0.552
BE2	−0.433	0.723	**0.956**	−0.396	−0.410	−0.538	−0.427	−0.569
BE3	−0.345	0.680	**0.951**	−0.305	−0.393	−0.460	−0.354	−0.486
BE4	−0.343	0.671	**0.920**	−0.311	−0.401	−0.435	−0.367	−0.463
CC1	0.773	−0.488	−0.347	**0.949**	0.476	0.739	0.740	0.699
CC2	0.841	−0.551	−0.404	**0.971**	0.536	0.749	0.818	0.705
CC3	0.812	−0.556	−0.391	**0.967**	0.536	0.716	0.785	0.721
CO1	0.596	−0.515	−0.441	0.556	**0.871**	0.498	0.601	0.572
CO2	0.421	−0.408	−0.362	0.413	**0.893**	0.333	0.444	0.424
CO3	0.373	−0.351	−0.320	0.412	**0.867**	0.292	0.392	0.383
DI1	0.701	−0.553	−0.495	0.700	0.371	**0.956**	0.707	0.704
DI2	0.757	−0.599	−0.532	0.757	0.448	**0.978**	0.724	0.721
DI3	0.775	−0.631	−0.551	0.763	0.467	**0.977**	0.737	0.742
DN1	0.763	−0.515	−0.362	0.724	0.506	0.669	**0.924**	0.624
DN2	0.835	−0.536	−0.416	0.784	0.544	0.723	**0.968**	0.683
DN3	0.848	−0.564	−0.448	0.810	0.555	0.734	**0.965**	0.701
DR1	0.711	−0.678	−0.580	0.672	0.513	0.714	0.685	**0.941**
DR2	0.665	−0.523	−0.451	0.655	0.502	0.612	0.611	**0.909**
DR3	0.710	−0.628	−0.519	0.728	0.492	0.748	0.669	**0.944**

**Table 4:** Factor loading and cross loading.

Variables	Defense of ubiquity (DU)	Intentions of compliance (IN)	Metaphor of the ledger (ML)	Normative belief (NB)	Cost of noncompliance (NC)	Response efficacy (RE)	Self-efficacy (SE)
DU1	**1.000**	−0.551	0.722	−0.468	−0.291	−0.482	−0.351
IN1	−0.541	**0.982**	−0.677	0.811	0.488	0.794	0.579
IN2	−0.543	**0.980**	−0.668	0.788	0.496	0.789	0.582
IN3	−0.536	**0.977**	−0.669	0.829	0.511	0.798	0.583
ML1	0.704	−0.666	**0.974**	−0.602	−0.323	−0.578	−0.355
ML2	0.708	−0.679	**0.984**	−0.616	−0.361	−0.597	−0.380
ML3	0.708	−0.666	**0.977**	−0.587	−0.345	−0.589	−0.337
NB1	−0.409	0.685	−0.499	**0.893**	0.473	0.642	0.524
NB2	−0.455	0.824	−0.616	**0.960**	0.506	0.738	0.582
NB3	−0.458	0.817	−0.613	**0.972**	0.524	0.746	0.604
NC1	−0.237	0.431	−0.291	0.433	**0.926**	0.405	0.312
NC2	−0.323	0.527	−0.368	0.536	**0.972**	0.478	0.361
NC3	−0.298	0.514	−0.358	0.529	**0.980**	0.464	0.365
NC4	−0.255	0.475	−0.323	0.534	**0.964**	0.453	0.353
RE1	−0.467	0.763	−0.571	0.713	0.465	**0.956**	0.638
RE2	−0.443	0.748	−0.535	0.693	0.412	**0.963**	0.628
RE3	−0.477	0.818	−0.619	0.763	0.474	**0.960**	0.624
SE1	−0.340	0.558	−0.330	0.578	0.382	0.613	**0.961**
SE2	−0.333	0.581	−0.352	0.601	0.319	0.650	**0.973**
SE3	−0.344	0.579	−0.376	0.577	0.353	0.637	**0.963**

**Table 5:** Goodness-of-fit measure of the model.

	*R* ^2^	Communality	Redundancy
Normative belief (NB)		0.888	
Response efficacy (RE)		0.921	
Cost of noncompliance (NC)		0.923	
Condemnation of condemners (CC)		0.926	
Self-efficacy (SE)		0.932	
Cost of compliance (CO)		0.770	
Intentions of compliance (IN)	0.816	0.960	0.336
Benefit of compliance (BE)		0.870	
Neutralization*	1	0.735	0.221
Denial of responsibility (DR)		0.868	
Appeal to higher loyalties (AL)		0.935	
Attitude (AT)	0.611	0.884	0.084
Defense of ubiquity (DU)		1	
Denial of injury (DI)		0.942	
Defense of necessity (DN)		0.907	
Metaphor of the ledger (ML)		0.957	

Average	0.809	0.901	0.214
Fitness of the model	0.857		

*Neutralization is a second-order construct.
